# Supplementary material for: Wolbachia and Asaia Distribution among Different Mosquito Vectors Is Affected by Tissue Localization and Host Species
Source: Microorganisms. 2024 Mar 8;12(3):545. doi: 10.3390/microorganisms12030545 (PMC10972390; doi:10.3390/microorganisms12030545)
Supplement: Supplementary file 1 [file microorganisms-12-00545-s001.zip › microorganisms-2900253-supplementary.pdf]

Wolbachia and *Asaia* distribution among different mosquito vectors is affected by tissue localization and host species.

Mahdokht Ilbeigi Khamseh Nejad[1,†,] Alessia Cappelli[2,†], Claudia Damiani[2], Monica Falcinelli[1], Paolo Luigi Catapano[1], Ferdinand Nanfack-Minkeu[3], Paul Audrey Mayi[1], Chiara Currà[4], Irene Ricci[2], Guido Favia[2,*]

Supplementary Tables

Table S1. Field collected mosquitoes included in the study.

Collection Years	Country	Village	Latitude	Longitude	Mosquito species	N°♂/N°♀
2008	Africa, Burkina Faso	Ouagadougou	12.3681873	-1.5270944	*Ae. aegypti*	7♀
					*An. funestus**	32♂/37♀
2022	Italy, Marche	Petriolo (Macerata)	43.221521	13.4663061	*Ae. albopictus**	72♀
	Italy, Marche	Camerino (Macerata)	43.1357641	13.0683092	*Cx. pipiens*	18♂/20♀
	Italy, Veneto	Pedavena (Belluno)	46.0396242	11.8804327	*Ae. albopictus*	9♀
					Ae. koreicus	3♀
					Ae. japonicus	4♀
					Cx. pipiens	2♀
		Sospirolo (Belluno)	46.1427254	12.0749943	*Ae. koreicus*	16♂/7♀
		Feltre (Belluno)	46.0163755	11.9062541	*Ae. koreicus*	3♂/2♀
					Ae. japonicus	16♂/18♀
	USA, Ohio	Wooster	40.798098	-81.939773	*Ae. japonicus*	18♂/21♀
	Africa, Cameroon	Yangah	10.860485	14.892721	*An. arabiensis*	37♀
					An. coluzzii	10♀
					An. pharoensis	48♀
					An. ziemanni	48♀
		Yaoundé	3.8689867	11.5213344	*An. coluzzii*	39♀
					An. gambiae	25♀
		Douala	4.0429408	9.706203	*An. coluzzii*	38♀
					An. gambiae	25♀
		Mbalmayo	3.515197	11.503114	*An. coluzzii*	34♀
					An. gambiae	5♀
		Santchou	5.281565	9.9760978	*An. gambiae*	45♀
		Adamaoua	6.8496708	13.2163222	*An. gambiae*	48♀
		Mbandjock	4.4461951	11.9024619	*An. gambiae*	41♀
		Dschang	5.4469923	10.053309	*Ae. albopictus*	44♀
2023	Italy, Veneto	Pedavena (Belluno)	46.0396242	11.8804327	*Ae. koreicus*	34♂/15♀
					Ae. japonicus	6♂/19♀
					Ae. albopictus	1♂/1♀
		Sospirolo (Belluno)	46.1427254	12.0749943	*Ae. koreicus*	4♂/6♀
					Ae. japonicus	5♂/15♀
					Ae. albopictus	3♂/9♀
		Feltre (Belluno)	46.0163755	11.9062541	*Ae. koreicus*	10♂/5♀
					Ae. japonicus	2♂/2♀
					Ae. albopictus	1♀
		Alano di Piave (Belluno)	45.907803	11.908195	*Ae. koreicus*	3♂/21♀
	Italy, Toscana	Pisa	43.7014507	10.4312849	*Cx. pipiens*	5♂/37♀
	Crete	Rethymno	35.3676472	24.4736079	*Ae. albopictus*	21♀
					Cx. pipiens	11♀
		Heraklion	35.33908	25.1332843	*Ae. albopictus*	8♀
					Cx. pipiens	9♀
		Hersonissos	35.308168	25.3720231	*Ae. albopictus*	26♀
					Cx. pipiens	22♀
		Gazi	35.3257586	25.0663826	*Ae. albopictus*	5♀

Supplementary Figures

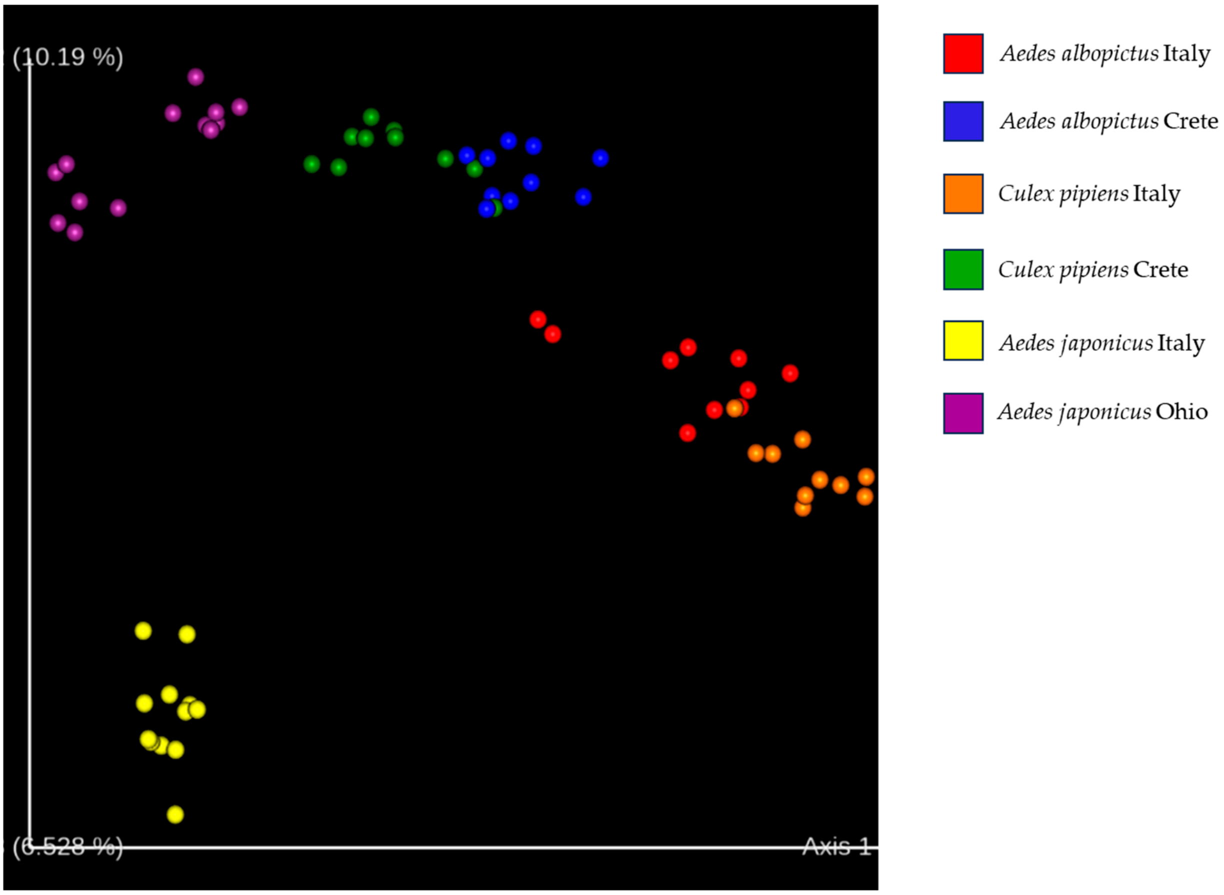

Figure S1: Principal Coordinates Analysis (PCoA) plots of samples coloured according of the sample sites. The comparison among the microbial composition were analyzed using Bray-Curtis-emperor method.
